# Fertility Sparing Treatment in Patients With Early Stage Endometrial Cancer, Using a Combination of Surgery and GnRH Agonist: A Monocentric Retrospective Study and Review of the Literature

**DOI:** 10.3389/fmed.2018.00240

**Published:** 2018-08-27

**Authors:** Stéphanie Tock, Pascale Jadoul, Jean-Luc Squifflet, Etienne Marbaix, Jean-François Baurain, Mathieu Luyckx

**Affiliations:** Department of Gynecology, Université catholique de Louvain, Cliniques Universitaires St Luc, Brussels, Belgium

**Keywords:** endometrial adenocarcinoma, conservative treatment, atypical endometrial hyperplasia, GnRH agonist, fertility-sparing

## Abstract

**Objectives:** To evaluate the efficacy and safety of gonadotropin-releasing hormone (GnRH) agonist after endometrial resection in women suffering early stage endometrial carcinoma (EC) and/or endometrial intra-epithelial neoplasia (EIN).

**Design:** A retrospective review of clinical files between January 1999 and December 2016.

**Setting:** University hospital.

**Patients:** Eighteen women younger than 41 years with grade 1 endometrial carcinoma (G1EC) and/or Endometrial intra-epithelial neoplasia (EIN). Interventions: All patients received GnRH agonist for 3 months after an endometrial resection combined with a laparoscopy to exclude concomitant ovarian tumor and/or other extra-uterine disease. The patient underwent a follow-up of 3 months interval with endometrial sampling by hysteroscopy.

**Main Outcome Measure(s):** The recurrence rate and the pregnancy rate after fertility sparing treatment.

**Results:** We identified 9 patients with EIN (50%), 7 patients with G1EC (38.9%), 1 with combined histology (5.5%), and 1 with G2EC (5.5%). After a median follow-up of 40.7 months, 12 patients conserved their uterus (66.7%), and 8 (53.3%) patients were pregnant with a total of 14 pregnancies among those who tried to become pregnant. We observed a complete response rate in 12 patients (66.7%) but 3 of these patients relapsed (25%). We also found a stable disease in 6 patients (33.3%).

**Conclusions:** Compared with other fertility sparing treatments, GnRH agonist after surgery is an effective fertility-sparing strategy for women with EIN and/or G1EC. We recommend hysterectomy once a family has been completed even if the literature does not clearly lead to radical surgery.

## Introduction

Endometrial cancer (EC) is the most common gynaecologic cancer in Western countries and its incidence has been steadily increasing in Eastern countries (1). Fourteen percent of EC are reported in premenopausal women and 5–29% occur before 40 years of age (2) with 70% of patients being nulliparous at the time of diagnosis (3).

In women of childbearing age, EC usually presents with favorable prognostic features that are: endometrioid histotype, focal and well-differentiated grade 1 (G1) lesion, no or minimal myometrial invasion, type 1 EC expressing high levels of estrogen receptor-alpha (ER) and progesterone receptor (PR) (4). In patients younger than 40 years, about 80% have stage I disease and 50–90% have grade I disease (5, 6).

Endometrial intra-epithelial neoplasia (EIN) is a precancerous lesion and 29% of such cases progress to EC within a few years (7). The most common identified risk factor for EC is obesity due to the peripheral conversion of androstenedione and androgens to estrogen (8). Among other identified risk factors, we note sedentary lifestyle, hyperinsulinemia and type 2 diabetes, hypertension, nulliparity, early menarche, Lynch syndrome, and anovulatory conditions such as polycystic ovarian syndrome (9).

The recommended treatment for patients diagnosed with EC is surgery with total hysterectomy, bilateral salpingo-oophorectomy (BSO), and possibly pelvic and para-aortic lymphadenectomy depending on the stage and the grade of the disease (4). The number of reproductive aged women who are delaying childbearing is increasing; therefore, it is important to provide them with a fertility-sparing option while providing them with a correct cancer treatment.

Fertility-sparing treatment (FST) is not a novel approach as Kistner showed in 1959 (10). In his publication, he reported 7 cases of endometrial hyperplasia and EIN who were not operated on but successfully treated with progestins with one patient obtaining a pregnancy.

**Figure 1:**
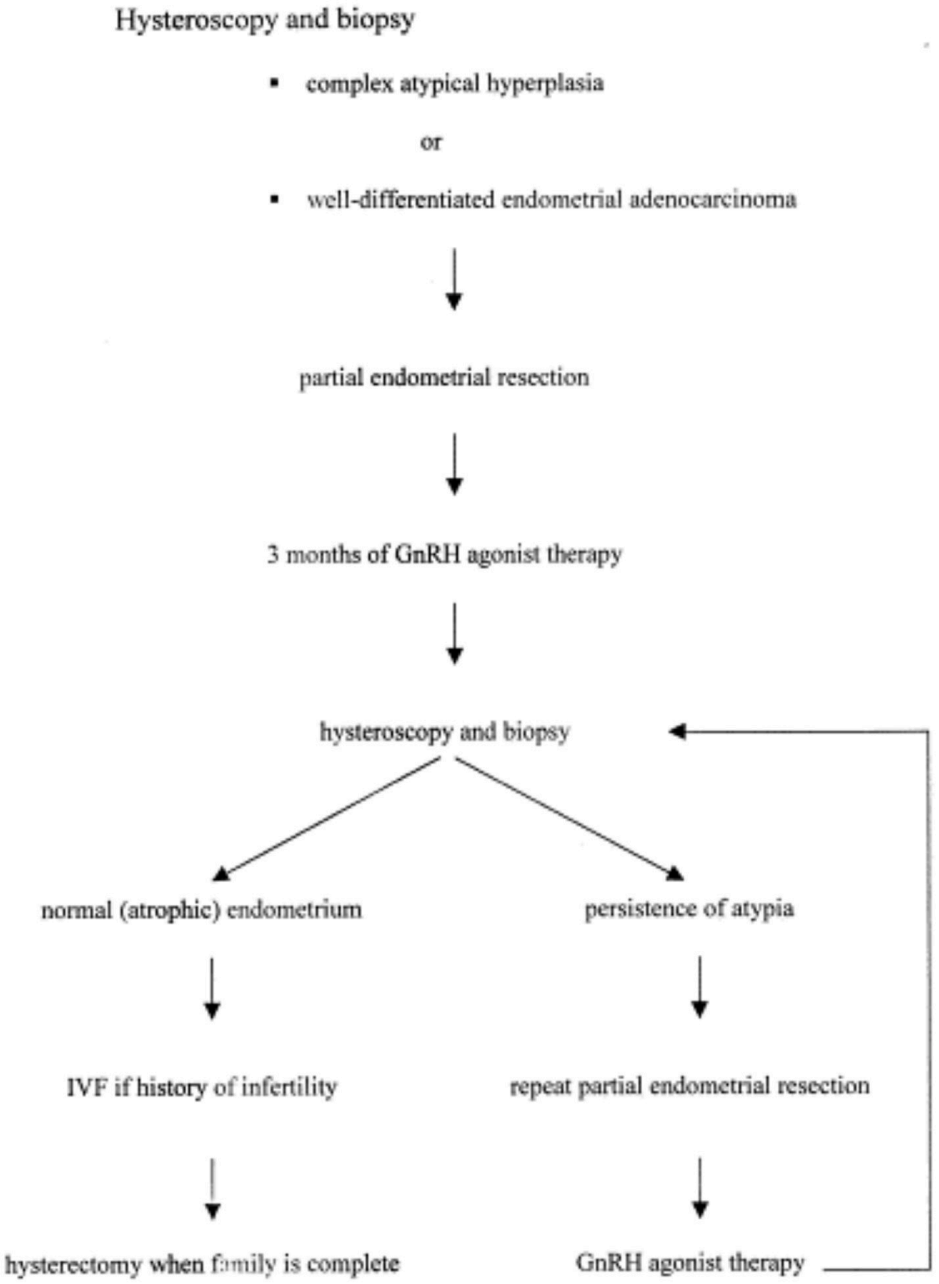
Conservative treatment in our department. Adapted from Jadoul and Donnez (11).

Today, the most common medical treatment is high-dose oral progestins such as medroxyprogesterone acetate (MPA), megestrol acetate (MA), or local high-dose progestins like levonorgestrel-releasing intra-uterine device (LNG-IUD). Other hormonal treatments have also been used including gonadotropin-releasing hormone (GnRH) agonist, hydroxyprogesterone, oral contraceptives, tamoxifen, and letrozole. In addition, there are some reports of surgical management. Therefore, a hysteroscopic resection of abnormal endometrium combined to GnRH agonist can be performed as Jadoul and Donnez demonstrated in 2003 (11), with good safety and fertility outcomes. Our department has been applying this fertility-sparing management for more than 10 years. The procedure is summarized in Figure 1.

As 11% of young patients with EC have synchronous associated cancer compared to 2% of those older than 45 years (12), a laparoscopic exploration of the abdomen has to be performed with biopsies of the ovaries in order to exclude any extra-uterine disease.

The importance of hysteroscopy in our opinion is that it offers the ability to diagnose and treat endometrial cancer by resecting the lesion under direct vision.

To date, the European Society of Gynaecological Oncology (ESGO) Task Force for Fertility Preservation study confirms that FST is a safe option for stage IA patients with endometrioid histotype and grade 1 EC (13).

## Materials and methods

We reviewed the clinical files of all the patients with EC treated in our institution between January 1999 and December 2016. Eligible patients were between 18 and 41 years, had histologically confirmed G1EC or EIN at presumed stage IA (according to the 1998 International Federation of Obstetrics and Gynaecology staging system), and desired to preserve their fertility despite an oncological risk. Eleven patients who were treated by hysteroscopic partial endometrium resection (tumor) and GnRH agonist therapy to maintain their fertility at Cliniques Universitaires Saint Luc between 2003 and 2016 were retrospectively analyzed.

Ethics committee of the Cliniques Universitaires Saint-Luc approved the study protocol.

We added this series to our previously published series in 2003 by Jadoul and Donnez (11). Altogether, 18 patients <42 years of age underwent conservative treatment of EC and/or EIN in our department since 1999.

Endometrial tissue sampling for diagnosis was carried out by hysteroscopy. All the macroscopically abnormal endometrium was removed by operative hysteroscopy, using monopolar energy. A diagnostic laparoscopy (including surface ovarian biopsies and peritoneal cytology) was incorporated in the enrolment work-up for all patients who were diagnosed with EC or EIN. Before the surgery, magnetic resonance imaging (14) was performed to exclude any myometrial infiltration. In case of clear myometrial infiltration, the patient was not offered to undergo FST.

Patients then received 3.6 mg of gosereline subcutaneously on a monthly (28 days) basis for 3 months or one dose of 10.8 mg of long-acting gosereline subcutaneously. At the end of the treatment, a diagnostic hysteroscopy with an endometrial biopsy was performed to assess the efficiency of GnRH agonist therapy.

Endometrial biopsy was performed under hysteroscopic vision or with Cornier's pipelle or Novak canula since it has been reported that pipelle biopsy and D&C show almost equal success rate in the diagnosis of endometrial pathologies (15) and pipelle biopsy is cheaper and less invasive to patients. Some patients underwent a biopsy without hysteroscopic diagnose.

Transvaginal sonography was routinely performed for each endometrial biopsy and endometrial thickness and the presence of extra-uterine disease (i.e., ascites, adnexal mass) were assessed.

The pathological response to GnRH agonist treatment was categorized as complete response (CR), partial response (PR), stable disease (16), or progressive disease (17) based on the previous report by Corzo et al. (9). CR was defined as the absence of any hyperplastic or neoplastic lesion. PR was defined as residual lesion down staged compared to the initial diagnosis. SD was defined as residual lesion identical to the pre-treatment lesion. PD was defined as the appearance or extension of endometrioid adenocarcinoma, of myometrial invasion or of any extra-uterine lesion. If the disease is progressive, a total hysterectomy with BSO was strongly recommended. Recurrence was defined as the appearance of EC or EIN during follow-up after an endometrial sample had shown disease regression. Time to recurrence was measured from the date of complete regression. Patients showing persistent, progressive, or recurrent disease were recommended to undergo definitive surgery.

If the lesion completely disappeared histologically, the patients were carefully monitored, and allowed to conceive. Patients underwent “diagnostic” hysteroscopy with endometrial biopsy every 3 months until they got pregnant.

If patients succeed to obtain a pregnancy pregnant and give birth, hysteroscopy and endometrial biopsy were performed every 3 months after delivery until another pregnancy or radical surgery was done. If the lesion recurred, a total hysterectomy plus BSO was generally recommended. When the patient considered her family complete or if she became too old for potential pregnancy, definitive surgery was then proposed. However, if the patient strongly preferred to preserve fertility, or refused definitive surgery, follow-up was continued.

The primary endpoints of this study were the pathologic CR rate, recurrence rate and obstetric outcomes.

Secondary endpoints were adverse events and cancer related deaths during the study periods. Adverse effects were evaluated according to Common Terminology Criteria for Adverse Events (18) v4.0.

## Results

Twenty patients aged 18–41 years with EC or EIN and wishing to preserve their fertility were retrieved from the clinical files, 7 of whom were already reported in 2003 (Jadoul and Donnez). Two out of the 13 further patients were excluded: one was lost for follow-up immediately after the diagnosis, and the other one was managed with a different fertility sparing treatment. One patient suffered of a grade 2 EC, but she refused a radical surgery because of her wish to conceive. We accepted her request after informing her of the risk she took and then performed the same FST.

Nine patients (50%) had a EIN, seven (38.9%) had G1EC, one (5.5%) patient had both types of lesions and one (5.5%) had G2EC. The median age was 32.5 (range: 18–41 years) and the median body mass index of the 11 last patients (this data was not recorded before 2003) was 25.6 kg/m^2^ (16.9–41.1). Only 2 patients were over 30 kg/m^2^. Sixteen patients were nulliparous. The median follow-up period was 40.7 months (5–180 months).

Sixteen patients had endometrial biopsy for diagnosis guided by hysteroscopy, two by curettage (D/C). All patients had a hysteroscopic resection together with laparoscopic evaluation. Before the resection, an MRI was systematically performed. None of the patient presented images of myometrium infiltration and/or extra uterine disease. The pretherapeutic laparoscopic evaluation didn't show any extra-uterine disease.

After the first hysteroscopic resection, all patients were given GnRH agonist for 3 months.

After completion of the treatment, patients underwent close follow-up including clinical examination, hysteroscopy with endometrial biopsies and transvaginal ultrasound (TVU). Among the 9 patients with EIN, 7 (78%) had a CR and 2 (22%) had a SD. Two patients with CR relapsed after 10 and 9 months (patients 4 and 5 respectively). Patient 4 underwent hysterectomy while patient 5 had a CR after a second endometrium resection followed by 3 months of GnRH agonist treatment. Among the 2 patients with SD, patient 6 underwent a second hysteroscopic resection combined with a three more GnRH agonist and the second one (patient 7) underwent a second hysteroscopic resection without injection of GnRH agonist. No intra uterine adhesion was noted in our series, even in the long-term follow-up carried on by hysteroscopy, which gave a regular direct view of the uterine cavity.

Among the 7 patients with G1EC, 4 had a CR (57%), and 3 (43%) had a SD. One of the patient with SD underwent a total hysterectomy, one was treated by a further endometrium resection and a further GnRH agonist treatment and the last one underwent a further endometrium resection. There was no relapse in the G1EC group.

The only patient who had the two types of lesions (EIN and G1EA) at the time of diagnosis had complete response but relapsed after 3 months and underwent hysterectomy.

The patient with G2EC underwent total hysterectomy with BSO after the first 3 months of follow-up because of the persistence of EIN (PR) at the control hysteroscopic biopsies.

No progressive disease was noted in our series.

Among a total of 12 CR (66%), 8 patients wished to become pregnant right away and 4 of them were pregnant (50%) including 3 with IVF. The 4 other patients underwent unsuccessful attempt of pregnancy and were followed during 64, 15, 32, and 19 months respectively (patient 1, 4, 5, and 14). Four patients did not plan to conceive right away and 3 were followed during 19, 7, and 62 months (patient 3, 8, and 10). The last one (patient 17) relapsed after 3 months, was treated by a second hysteroscopic endometrium resection and because of EIN at a further followed biopsy, she underwent hysterectomy.

Among the 6 patients with SD, 4 (67%) got pregnant with a total of 5 live births. The other 2 patients underwent hysterectomy because of the SD (G1EC for patient 11 and EIN for patient 18). On the entire cohort, 8 patients (44%) obtained one or more pregnancies, with 11 live births and 4 did not try. Four (22%) patient did not succeed to obtain a pregnancy even if they had conserved her fertility, with one of her who need to undergo an hysterectomy for recurrent disease.

Among all the 18 patients, 6 (33%) underwent hysterectomy as final treatment including 2 patients once the family had been completed (patients 2 and 6), meaning that only 4 (22%) underwent hysterectomy because of failure of FST: one because of recurrent disease after 10 months (patient 4), two because of SD or PR respectively (patients 11 and 18) and one because of SD 3 months after a second FST for a recurrent disease (patient 17).

Pregnancies occurred after an average of 3.5 months (range 0–20 months) after the end of GnRH agonist therapy. This time seems to be shorter than in the literature. An explanation could be the median age in our population was lower than other studies.

The median follow-up is 40.7 months, and 12 (66.7%) patients still have their uterus today. No patient had progressive disease and none died in our series. However, some patients had shorter follow-up, that could bias our recurrence rate results. The follow-up times for patients 12 and 13 were 14 and 15 months respectively with only 2 endometrial biopsies (at 3 months post-GnRH agonist and 3 months post-delivery). They got pregnant with IVF directly following the 3 months treatment with GnRH agonist and were followed respectively during 2 and 3 months after delivery.

**Figure 2:**
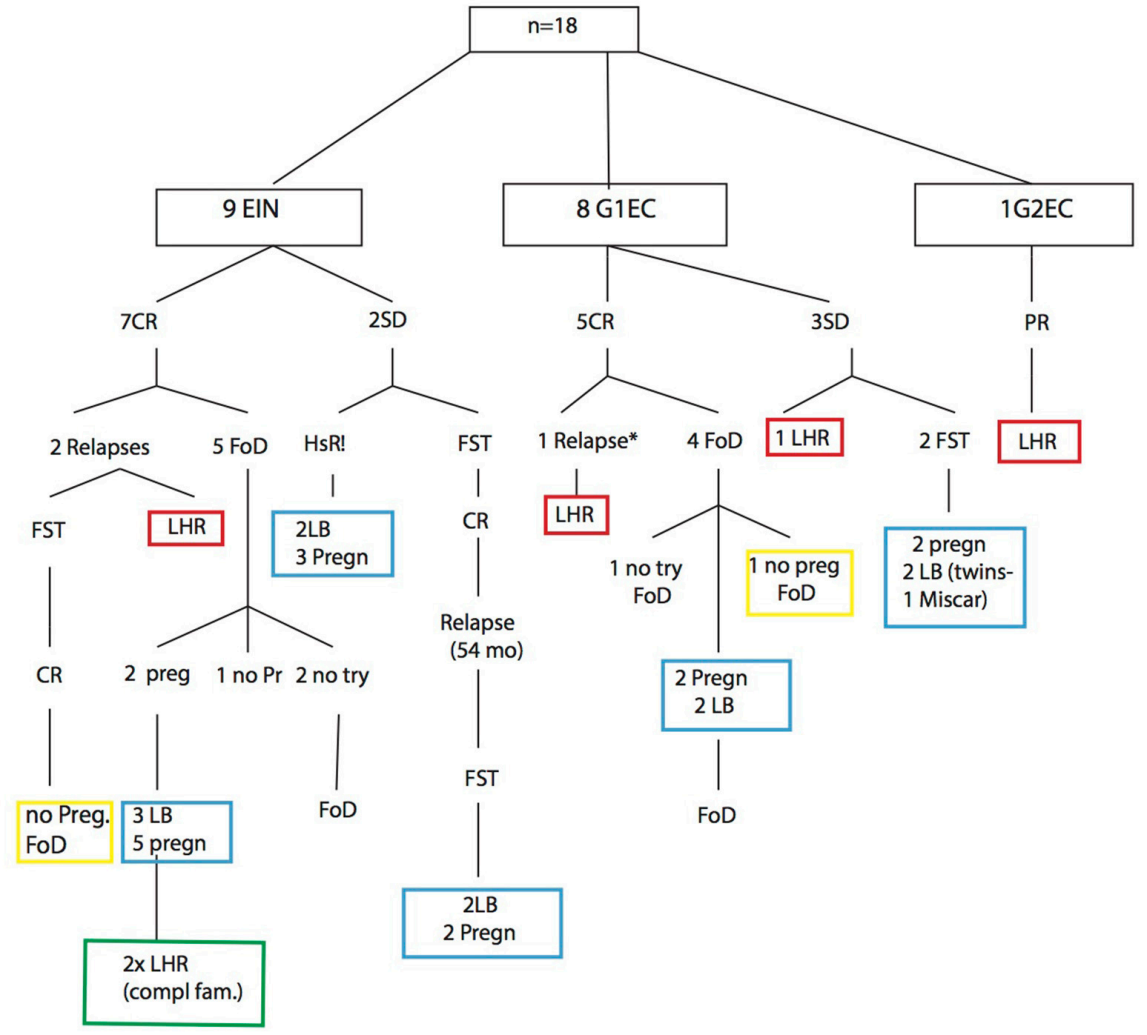
Flow-chart and summary of results.

Results are summarized in Table 1 and in Figure 2.

**Table 1:** Personal experience-results.

	**Age**	**Parity**	**Infertility**	**Diagnose**	**Histo-pathology**	**Treatment**	**Response**	**Treatment if SD**	**Relapse**	**Treatment if relapse**	**Pregnancy**	**Life birth**	**Stimulation**	**Final LHR**	**Indication of LHR**	**Follow-up**
1	39	1	5 y	HB	CAH/EIN	HSR+aGnRH 3 m	CR				None			N		64
2	37	0	none	HB	CAH/EIN	HSR+aGnRH 3 m	CR				4	2		Y	Completed family	91
3	31	0	1 y	HB	CAH/EIN	HSR+aGnRH 3 m	CR				Not try			N		19
4	41	0	1 y	HB	CAH/EIN	HSR+aGnRH 3 m	CR		10 m	LHR	None			Y	Relapse	15
5	28	0	1 y	HB	CAH/EIN	HSR+aGnRH 3 m	CR		9 m	HSR+aGnRH 3 m	None			N		32
6	27	0	None	D/C	CAH/EIN	HSR+aGnRH 3 m	SD	HSR+aGnRH 3 m	54 m	HSR+aGnRH 3 m	2	2		Y	Completed family	180
7	34	0	6 m	HB	CAH/EIN	HSR+aGnRH 4 m	SD	HSR			3	2	IVF	N		69
8	38	0	10 y	HB	CAH/EIN	HSR+aGnRH 3 m	CR				Not try			N		7
9	34	0	5 y	HB	CAH/EIN	HSR+aGnRH 3 m	CR				1	1	IVF	N		48
10	18	0	None	D/C	G1EC	HSR+aGnRH 6 m+Primolut®	CR				Not try			N		62
11	31	0	>1 y	HB	G1EC	HSR+aGnRH 3 m	SD	LHR			None			Y	SD	5
12	27	1	2 y	HB	G1EC	HSR+aGnRH 3 m	CR				1	1	IVF	N		14
13	28	0	None	HB	G1EC	HSR+aGnRH 3 m	SD	HSR+aGnRH 3 m			1	2	IVF	N		15
14	38	0	4 y	HB	G1EC	HSR+aGnRH 3 m	CR				None		IVF	N		19
15	37	0	16 y	HB	G1EC	HSR+aGnRH 4 m	SD	HSR			1	0	IVF	N		10
16	37	0	6 y	HB	G1EC	HSR+aGnRH 3 m	CR				1	1	IVF	N		72
17	27	0	None	HB	CAH/EIN+G1EC	HSR+aGnRH 3 m	CR		3 m	HSR	None			Y	Persistent CAH/EIN after treatment for relapse	5
18	34	0	2 y	HB	G2EC	HSR+LNG-iud+aGnRH 3 m	SD	LHR			None			Y	SD	6

*HB, hysteroscopic biopsy; D/C, dilatation/curettage; HSR, hysteroscopic resection; LNG-iud, levonorgestrel intra-uterine device; LHR, laparoscopic radical hysterectomy; pp, post-partum*.

## Discussion

We conducted a retrospective study of all patients treated with FST using GnRH agonist in our institution and showed that women under 41 years with EIN and G1EC could be successfully managed by this strategy, with a failure rate of 22% and a pregnancy rate of 53.3% among the patients who tried to become pregnant. CR rate after the first round of treatment is 66.6% but considering all the patient that finally obtained CR we rise to 16 (89%) patient that were allowed to conserve their uterus, making our series in the higher CR rate regarding the literature.

Four patients were pregnant in the CR group and all the patients who had planned to conceive in the SD group were pregnant. Among the EIN group, 2 patients with SD (patient 6 and 7) got pregnant and each one gave birth twice. In the G1EC group, two thirds of the patients with CR got pregnant among those who tried to be. The same rate of pregnancy (2/3) was found with SD. After a median follow-up of 40.7 months, 12 out of 18 patients (66.7%) had their uterus.

Table 2 shows a summary of the 14 studies reporting outcome of patients with EIN or G1EC treated with FST from 2009 to 2017. A total of 516 patients were treated but because Inoue et al. (7) did not study the CR rate, we considered only 418 patients. Three hundred thirty-two patients experienced CR (79.4%), varying between 54.5 and 100% according to the study. The recurrence rate varied between 0 and 62% and the pregnancy rate between 25 and 93.3%. Our data are similar to those results with a CR of 67% (12/18), but rising 89% considering the CR after multiple FST, a recurrence rate of 25% (3 on the 12 patients with CR), and a pregnancy rate of 53.3%.

**Table 2:** Review of literature.

		**Treatment**	**CR**	**Recurrence**	**Pregnancy rate**	**LB**
2016	Inoue et al. (7)	MPA	NA	61/98 = 62%	45/9 = 45.9%	NA
2014	Ohyagi-hara et al. (19)	MPA	20/27 = 74%	9/20 = 45%	5/13 = 38.4%	9/13 = 69.2%
2014	Simpson et al. (21)	MPA	24/44 = 54.5%	13/24 = 54.2%	5/11 = 45.5%	2/11 = 18.2%
2013	Park et al. (3)	MPA or MA	115/148 = 77.7%	35/115 = 30.4%	44/115 = 38.3%	NA
2009	Mao et al. (15)	MPA or MA	4/6 = 66.6%	0/4 = 0%	$\frac{3}{4}$ = 75%	NA
2015	Mitsuhashi et al. (20)	MPA + metformin	29/36 = 80.6%	3/29 = 10.3%	8/16 = 50%	6/16 = 37.5%
2011	Kim et al. (22)	MPA + LNG-iud	4/5 = 80%	0/4 = 0%	NA	NA
2015	Wang et al. (5)	HSR + MA	6/6 = 100%	0/6 = 0%	3/6 = 50%	3/6 = 50%
2013	Shan et al. (25)	HSR + MA	21/26 = 80.8%	6/21 = 28.5%	2/8 = 25%	1/8 = 12.5%
2015	De Marzi et al. (24)	HSR + MPA	23/23 = 100%	1/23 = 4.3%	6/23 = 26.1%	5/23 = 21.7%
2010	Mazzon et al. (2)	HSR + MA	6/6 = 100%	0/6 = 0%	4/6 = 66.7%	5/6 = 83.3%
2017	Falcone et al. (4)	HSR + MA or LNG-iud	25/28 = 89.3%	2/26 = 7.7%	14/15 = 93.3%	13/15 = 86.6%
2010	Minig et al. (6)	LNG-iud + aGnRH	27/34 = 79.4%	6/27 = 22.2%	10/27 = 37%	7/27 = 25.9%
2017	Zhou et al. (23)	LNG-iud or letrozole + aGnRH	27/29 = 93.1%	2/27 = 7%	NA	NA
2018	Present study	HSR + aGnRH	12/18 = 66.6%	3/12 = 25%	8/15 = 53.3%	11/15 = 73.3%

*Abbreviations: MA, megestrol acetate; MPA, medroxyprogesteron acetate; HSR, hysteroscopic resection; LNG-iud, levonorgestrel intra-uterin device; CR, complete response; LB, life birth*.

Pregnancy rate = number of pregnancy among those who tried to be pregnant

*Life birth rate = number of life birth among those who tried to be pregnant*.

Among these 14 studies, 7 only used MPA or MA as FST (3, 7, 15, 19–22) and 2 combined GnRH agonist and LNG-IUD (6, 23) whereas 5 used hysteroscopic resection combined to high-dose oral progestin (2, 4, 5, 24, 25). As far as we know, no other study [except Jadoul and Donnez (11)] reported the effect of GnRH agonist combined with hysteroscopic endometrium resection as FST for EIN or EC. Therefore, majority of studies use high-dose of progesterone as FST (MPA or MA). The results of these treatments are heterogeneous. Indeed, the CR rate varied between 54.5% (21) and 80.6% (20) and the pregnancy rate between 38.3% (3) and 75% (15). There was no difference in the CR rate between these two drugs (3). One of them used metformin combined with progestin (20). This study supported the notion that metformin use reduces the recurrence rate and offers a protective effect in terms of EC development.

The problem with progesterone is that it has many side effects including changes in appetite, weight gain, fluid retention, acne, headache, depression, liver injury, breast discomfort, or irregular bleeding. Regarding the risk of venous thrombo-embolism (VTE), the majority of studies did not suggest an increase in odds for venous or arterial events with progesterone treatment. However, a few studies suggested an increase of VTE with use of injectable progestins for therapeutic indications (26). One of them suggested that the odds of VTE are higher among smokers using high-dose of progestin compared with smokers who did not use this hormonal treatment (27). Another study showed significantly elevated odds of VTE among women with factor V Leiden who used MPA comparing with non-use (28). Christiansen et al. noted an elevated chance of recurrent VTE among women with a history of VTE using high-doses of progestin but that did not reach statistical significance (29).

The side effects which could be reported in our review was weight gain (15, 25), liver dysfunction (20, 25), swelling or pigmentation of the face and neck (25) or diarrhea and nausea because of metformin (20).

The alternative to oral systemic progestin is the LNG-IUD, which provides very high-doses of progestin to the local endometrium and avoids the systemic effects produced by oral progestin. In our review, we didn't find a study which use LNG-IUD alone as FST. Kim et al. (22) performed a prospective observational study with MPA and LNG-IUD as FST. Some studies reported a FST combining LNG-IUD and GnRH agonist or progestin therapy as described in a recent meta-analysis (30) wherein a CR, a pregnancy rate and a recurrence rate of 72.9, 56, and 11% were shown respectively among the studies which use this FST.

Among the two studies using only combination of GnRh agonist and LNG-IUD, no major adverse effect was reported but only symptoms of discomfort such as hot flashes and vaginal dryness (6, 23). The CR was 79.4% (6) and 93.1% (23), the recurrence rate was 22.2% (6) and 7% (23). About pregnancies, Minig et al. reported a rate of 37% (6). Zhou et al. (23) did not give the pregnancy rate. Therefore LNG-IUD could be used in combination with GnRH agonist. Other team used aromatase inhibitors (AIs) with the GnRHa (31). IAs can reduce the levels of estrogen by inhibiting estrogen synthesis which leads to a reduction in the receptor-mediated growth stimulated in hormonal-dependant cancer such as EC (23). Using AIs seems to be an alternative FST for the obese women who failed to respond to oral progesterone or LNG-IUD (32). That can be explained by the peripheral conversion of androgens to estrone in adipose tissue that leads to high levels of serum estrogen.

Concerning hysteroscopic resection combined with progestin, the results in the literature seem to suggest an additional advantage in terms of CR rate and incidence of successful pregnancy (2). This pattern is confirmed by the recent publication of Fan et al. (30) with a CR of 95.3%, a pregnancy rate of 47.8% and a recurrence rate of 14.1%. No serious toxic side-effects occurred barring liver dysfunction in one patient (5).

Regarding GnRH agonist, the most serious side effect is the risk of bone loss (33). However, bone loss is minimal if the treatment does not exceed 3–4 months. A 6-month therapy appears to be associated with a decrease of up to 8.2% in lumbar bone density (33). Other side effects to consider with GnRH agonist are menopause-like symptoms such as vaginal dryness, hot flushes, reduced sexual interest, insomnia, headache, depression, nausea and vomiting. Those symptoms seem to be intolerable for about 10% of patients (34), but some of these can be reduced with tibolone (35, 36).

According to our results, GnRH agonist with hysteroscopic resection can be used as an alternative treatment to a high dose of progestin and LNG-IUD with some advantages. Indeed, GnRH agonist injection prevents the suboptimal compliance of oral progestin treatment and does not increase the risk of thrombophlebitis, change in lipid metabolism, atherogenesis, or other diseases such as diabetes, hypertension, or hyperlipoproteinemia (37, 38).

On the other hand, GnRH agonist seems to play an important role in the maintenance of intra-uterine tissues and the development of endometrial cancer (2, 4, 19, 20). Thus, GnRH agonists may have an antiproliferative effect in the growth of endometrial cancer cells by directly regulating the tumor progression (21). According to Wu et al. (39), the potential role of GnRH in promoting the cell migration and invasion of endometrial cancer is through the binding of GnRH-I receptors, the activation of the ERK1/2 and JNK pathways, and the subsequent induction of the metastasis-related matrix metalloproteinase-2 activity.

Adding endometrium resection to the hormonal treatment increases the risk of intrauterine adhesion, which represents the major long-term complication of operative hysteroscopic procedures, with an incidence that varies according to type and extension of surgery, surgical indication and patient's age (24). In order to keep a maximum of normal endometrium to allow pregnancy, the endometrium resection must not be too deep in the normal-looking part of the cavity (blind biopsies) and a complete resection must be limited to the macroscopically abnormal endometrium. Endometrium resection has to be performed under anesthesia (general or loco-regional) to permit a bipolar or monopolar resection without pain and in good surgical conditions. In our series, no intra uterine adhesion was noted. In contrast with curettage, hysteroscopic resection may increase the therapeutic efficacy by excising the tumor under direct vision. However, Falcone et al. showed the curettage is associated with the lowest rate (<10%) of histological under-grading (4).

Patient evaluation before treatment is a crucial point in a conservative approach.

Our protocol for FST includes a pre-treatment evaluation by MRI and laparoscopic exploration to exclude a synchronous ovarian cancer, even if this pre-treatment assessment is not a common procedure in the literature. The rate of coexisting ovarian malignancies and endometrial cancer in young women (<45 years) varies between 5 and 29% (12, 40–43). Therefore, this pre-treatment procedure is an essential step in the evaluation of endometrial lesion as suggested by several authors (44, 45). In our retrospective series, no ovarian malignancies were found.

As routine follow-up, we performed a diagnostic hysteroscopy (“no-touch” technique) with biopsy every 3 months. In case of pregnancy, this routine follow-up restart 3 months after delivery.

Because of the relatively high recurrence rate, we recommend that patients start trying to get pregnant directly after treatment. In our study, 8 patients had one or more pregnancies. A total of 14 pregnancies were obtained with a live birth rate of 11/14 (78.5%). Seven pregnancies have been achieved with IVF yielding 6 live births. Time to becoming pregnant is on average 3.5 months.

One of the limitations of our study was the retrospective nature of the work. These data need to be confirmed in a prospective multi-institutional study to explore the possibility of a resectoscopic management combined with GnRH agonist for treating EIN or G1EC in women who wish to preserve their fertility. Given the small number of patients, the clinical value of the combined therapy of early-stage endometrial cancer may not be conclusive and should be verified by further studies using larger sample sizes. Although the present sample is small, it is encouraging enough to support continuation of this FST. In the future, we could evaluate hormone receptor status, particularly the expression of estrogen and progesterone receptors.

Another issue may be the short follow-up for some patients who went to a different institution after pregnancy.

Unfortunately, there is no definitive consensus regarding optimal patient selection, medical treatment or surgical treatment nor about the treatment duration, follow-up schedule and the best time for definitive therapy. The dose and duration of treatment are still not standardized (4).

In conclusion, we believe that hysteroscopic resection combined to GnRH agonist is appropriate for evaluating the disease and a safe and efficient FST due to the low rate of recurrence and the absence of progressive disease with a good rate of long term uterine preservation and high pregnancy rate. Radical surgery can be proposed as definitive treatment in case of SD or recurrence and is recommended once the family is completed. The risk of intra-uterine adhesions seems to be very low and none were found in our series.

## Author contributions

ST is the author who has written the manuscript and collected all the data. PJ, J-FB, EM, and J-LS participated actively in drafting sections of the manuscript, editing and approving the final submitted version. ML is the main supervisor of this work and he revised the final article.

### Conflict of interest statement

The authors declare that the research was conducted in the absence of any commercial or financial relationships that could be construed as a potential conflict of interest.
